# Manganese Toxicity Inhibited Root Growth by Disrupting Auxin Biosynthesis and Transport in *Arabidopsis*

**DOI:** 10.3389/fpls.2017.00272

**Published:** 2017-03-03

**Authors:** Jingjing Zhao, Wenying Wang, Huakun Zhou, Ruling Wang, Ping Zhang, Huichun Wang, Xiangliang Pan, Jin Xu

**Affiliations:** ^1^Key Laboratory of Tropical Plant Resources and Sustainable Use, Xishuangbanna Tropical Botanical Garden, Chinese Academy of Sciences, KunmingChina; ^2^College of Life Science and Geography, Qinghai Normal UniversityXining, China; ^3^Northwest Institute of Plateau Biology, Chinese Academy of SciencesXining, China; ^4^College of Environment, Zhejiang University of TechnologyHangzhou, China

**Keywords:** manganese (Mn) toxicity, auxin accumulation, auxin efflux carriers, primary root growth, *Arabidopsis*

## Abstract

Mn toxicity inhibits both primary root (PR) growth and lateral root development. However, the mechanism underlying Mn-mediated root growth inhibition remains to be further elucidated. Here, we investigated the role of auxin in Mn-mediated inhibition of PR growth in *Arabidopsis* using physiological and genetic approaches. Mn toxicity inhibits PR elongation by reducing meristematic cell division potential. Mn toxicity also reduced auxin levels in root tips by reducing IAA biosynthesis and down-regulating the expression of auxin efflux carriers *PIN4* and *PIN7*. Loss of function *pin4* and *pin7* mutants showed less inhibition of root growth than *col-0* seedlings. These results indicated that this inhibitory effect of Mn toxicity on PR growth was mediated by affecting auxin biosynthesis and the expression of auxin efflux transporters PIN4 and PIN7.

## Introduction

Manganese (Mn) is one of the essential micronutrient elements that constitutes the Mn-SOD and Mn-cluster of the oxygen-evolving complex in photosystem II and is also involved in the biosynthesis of acyl lipids, flavonoids, and lignin (Lidon et al., 2004). Therefore, Mn is necessary for plant growth and development (Chen et al., 2015). However, excess Mn is toxic to plants. Manganese (Mn) toxicity is probably the most important growth-limiting factor after aluminum (Al) for plants in acidic soils in some subtropical and tropical areas. Mn toxicity induced oxidative damage and thereby disrupting photosynthesis system in leaves. Excess Mn results in a rapid accumulation of reactive oxygen species (ROS) in plants and then causes chlorosis and necrosis in leaves (Le Bot et al., 1990). Srivastava and Dubey (2011) found that treatment with high concentration of Mn (3 and 6 mM MnCl_2_) induce oxidative stress, lowers the pool of antioxidants and elevates activities of antioxidative enzymes in rice seedlings. Excess Mn (0.5 or 1.5 mM MnCl_2_) inhibited photosynthetic efficiency in the long term hydroponic-cultured *Arabidopsis* plants (Millaleo et al., 2012). Mn toxicity disrupted thylakoid structure and the photosynthetic electron transport chain, thus it is possible that chloroplast is the primary target of Mn toxicity (Lidon et al., 2004; Chen et al., 2015).

Roots are also the primary target of Mn toxicity in plants. Roots of the plants exposed to Mn toxicity also exhibited a reduction in root growth, browning, and cracks in the roots (Le Bot et al., 1990). Most studies on the physiological mechanisms of plant responses to Mn toxicity were focused on Mn-inhibited photosynthesis, Mn-mediated changes in antioxidative enzymes, and ROS production. However, the studies on root physiology of plant responses to Mn toxicity are rare.

Auxin plays an important role in modulating root growth and responding to circumstance cues in plants. The AUXIN1/LIKE AUX1 (AUX1/LAX) family and the efflux carriers of the PINFORMED (PIN) family are required for optimal auxin distribution in root tips by regulating polar auxin transport (PAT) and are vital for modulating root growth and development (Laskowski et al., 2008; Yuan et al., 2013; Li et al., 2015; Shi et al., 2015). Mutations in these carrier genes result in dramatic defects in the root system architecture (RSA) (Laskowski et al., 2008; Shi et al., 2015). The Aux/IAA proteins are the negative regulators of auxin signaling (Ouellet et al., 2001). Modulation of Aux/IAA stabilization represents a general response strategy in plants to environmental cues (Yuan and Huang, 2016). Exposure to a high concentration of copper (Cu) inhibited primary root (PR) elongation by repressing PIN-FORMED 1 (PIN1) protein expression, thereby disrupting auxin transport in roots (Yuan et al., 2013). Cadmium (Cd) toxicity-induced decrease in auxin level and response in root tips is due to reduced PIN1/3/7 accumulation and increasing IAA17 stabilization (Yuan and Huang, 2016). AUX1 and PIN2 protect lateral root (LR) formation during the early stages of iron (Fe) stress in *Arabidopsis* (Li et al., 2015). Al toxicity inhibited root growth by modulating the expression and localization of PIN2 in roots (Shen et al., 2008; Wu et al., 2014). Zhu et al. (2013) found that auxin negatively regulates Al toxicity tolerance. Al toxicity induced auxin accumulation in the transition zone (TZ) of the root tips by *TAA1*-regulated local auxin biosynthesis in root tip TZ (Yang et al., 2014). However, whether and how Mn toxicity affects root growth by mediating auxin distribution and response in roots remains poorly understood.

In this study, we characterized Mn-inhibited root growth and described altered patterns of auxin accumulation and distribution in the PR of Mn-treated roots. Our results indicated that Mn toxicity reduced auxin levels in root tips by reducing IAA biosynthesis and down-regulating the expression of auxin efflux carriers PIN4 and PIN7 in *Arabidopsis*. The potential mechanisms involved in this process were discussed.

## Materials and Methods

### Plant Materials and Growth Conditions

Wild-type *Arabidopsis col-0* was used in this study. The following mutant and transgenic *Arabidopsis* lines were used: *pin4-3*, *pin7-2*, and *DR5:GUS*, *DII-VENUS*, *PIN1:PIN1-GFP*, *PIN2:PIN2-GFP*, *PIN4:PIN4-GFP*, *PIN7:PIN7-GFP*, *AUX1:AUX1-YFP*, *CYCB1;1:GUS*. *Arabidopsis* seeds were surface sterilized with 50% (v/v) bleach for 5 min and then rinsed with sterile deionized water five times. Sterilized seeds were sown onto 1/2 Murashige and Skoog (MS) agar medium [Sigma-Aldrich; supplemented with 1% (w/v) agar and 1.5% (w/v) sucrose, pH 5.75], and incubated for 2–3 days at 4°C in the dark to synchronize germination. The seedlings were grown vertically for 5 days in standard aseptic growth conditions at 22°C with a 16 h light/8 h dark photoperiod. Five-day-old seedlings were transferred onto plates supplemented with MnCl_2_ for 1–3 days.

### GUS Staining and Measurement of Fluorescence Microscopy

Seedlings harboring the GUS reporter gene were incubated at 37°C for 2–3 h in GUS staining solution with the substrate 1 mM X-Gluc (5-bromo-4-chloro-3-indoxyl-beta-D-glucuronic acid cyclohexylammonium salt), and then discolored in 95% (v/v) ethanol before microscopic examination (Zeiss Axioskop).

The GFP lines were observed with a confocal laser scanning microscope (Zeiss) according to the manufacturer’s instructions. The excitation and emission wavelengths were 488 and 520 nm, respectively. The roots treated with NO-specific fluorescent probe DAF-2 DA (Beyotime, China) were visualized using fluorescence microscope (Zeiss, excitation wavelength at 488 nm and emission wavelength at 525 nm).

### Phenotypic Analysis

To investigate the effects of Mn toxicity on root growth and development, 5-day-old *Arabidopsis* seedlings grown in 1/2 MS medium were transferred to fresh media containing 2–12 mM MnCl_2_ for 2 days. The relative root length was calculated as the PR length grown in the treatment conditions divided by the mean PR length under control conditions (Freeman et al., 2010). At least 60 seedlings were analyzed for each treatment.

### qRT-PCR Analysis

Five-day-old *Arabidopsis col-0* seedlings grown vertically on 1/2 MS medium were transferred onto 1/2 MS medium supplemented with 4 mM MnCl_2_ for 2 days. Total RNA was isolated from seedlings (100 mg) using RNAiso Plus (TaKaRa) according to the manufacturer’s instructions. The concentration of RNA was quantified spectrophotometrically using a NanoQuant. Reverse transcription was performed using the PrimeScript^TM^ RT Reagent Kit with gDNA Eraser (TaKaRa). SYBR-green quantitative RT-PCR was performed with Platinum^®^ SYBR^®^ Green qPCR SuperMix-UDG (Invitrogen). *ACTIN2* (AT3G18780) and *EF1a* (AT5G60390) were used as internal controls for qRT-PCR normalization using GeNorm (Czechowski et al., 2005). The gene-specific primers are presented in Supplementary Table S1. All primer pairs produced only one peak in DNA melting curves indicating high specificity of the primers. Three independent biological replicates and three technical repetitions were performed for each gene.

### Statistical Analysis

For the PR growth, GUS staining and fluorescence microscopy analysis, the experiments were repeated three times with at least 20 roots in each repeat. The experiment of nutrient content analysis was repeated six times. The data were analyzed using Image Pro Plus software (version 4.5.1.29; Media Cybernetics, Carlsbad, CA, USA) and SPSS (Statistic Package for Social Science) software. The results are presented as mean ± SD of three (or six) independent experiments. For statistical analysis, we used Tukey’s test.

## Results

### Mn Toxicity Inhibited PR Growth

**FIGURE 1:**
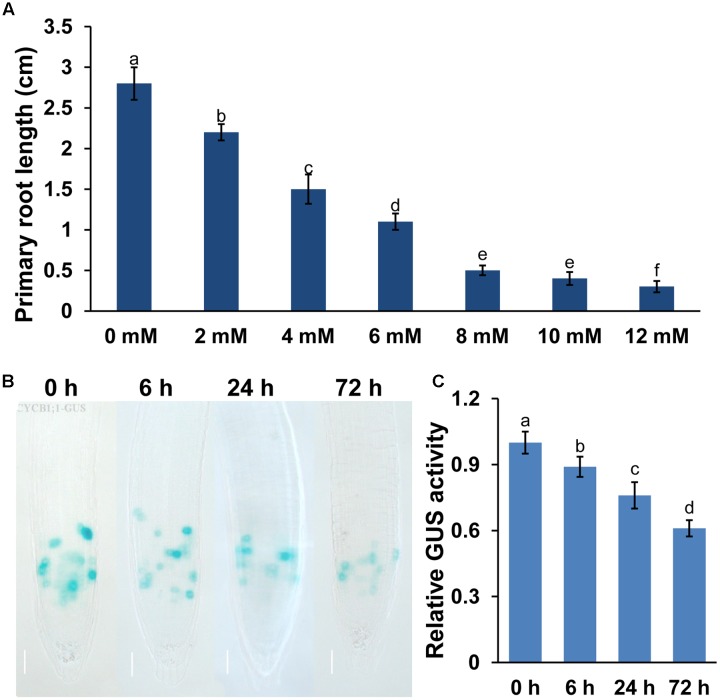
**Manganese (Mn) toxicity inhibited primary root (PR) growth in *Arabidopsis*.** Five-day-old *Arabidopsis* seedlings grown in 1/2 MS medium were transferred to fresh media containing 2–12 mM MnCl_2_ for 2 days. **(A)** The PR length was measured after 2 days of treatment. Image of GUS staining **(B)** and the relative GUS activity **(C)** of 5-day-old *CYCB1;1:GUS* seedlings exposed to 4 mM MnCl_2_ for 6, 24, and 72 h. Different letters indicate that they were significantly different at *P* < 0.01 by Tukey’s test. Error bars represent the ±SD.

Mn toxicity inhibited plant growth and development. In this study, we would like to explore the mechanisms for Mn-induced PR growth inhibition. Thus, we first tested the effects of different concentration of Mn on PR growth. As shown in **Figure 1A**, the growth of PR was significantly reduced by exposure to excess Mn, and the reduction of PR elongation correlated positively with Mn concentrations. Because the treatment with 4 mM MnCl_2_ induced an approximately 50% decrease of PR growth, we thus selected this concentration in subsequent experiments.

The results above showed that Mn toxicity inhibited PR growth. We then examined whether Mn toxicity repressed root growth and development by modulating the meristematic cell division potential by using the transgenic lines *CYCB1;1:GUS* (a marker used to monitor cell cycle). Mn toxicity significantly repressed the GUS activity in *CYCB1;1:GUS* (**Figures 1B,C**) seedlings. These data indicated that Mn toxicity inhibited root growth by reducing the meristematic cell division potential in root tips.

### Mn Toxicity Reduced Auxin Accumulation in Root Tips

**FIGURE 2:**
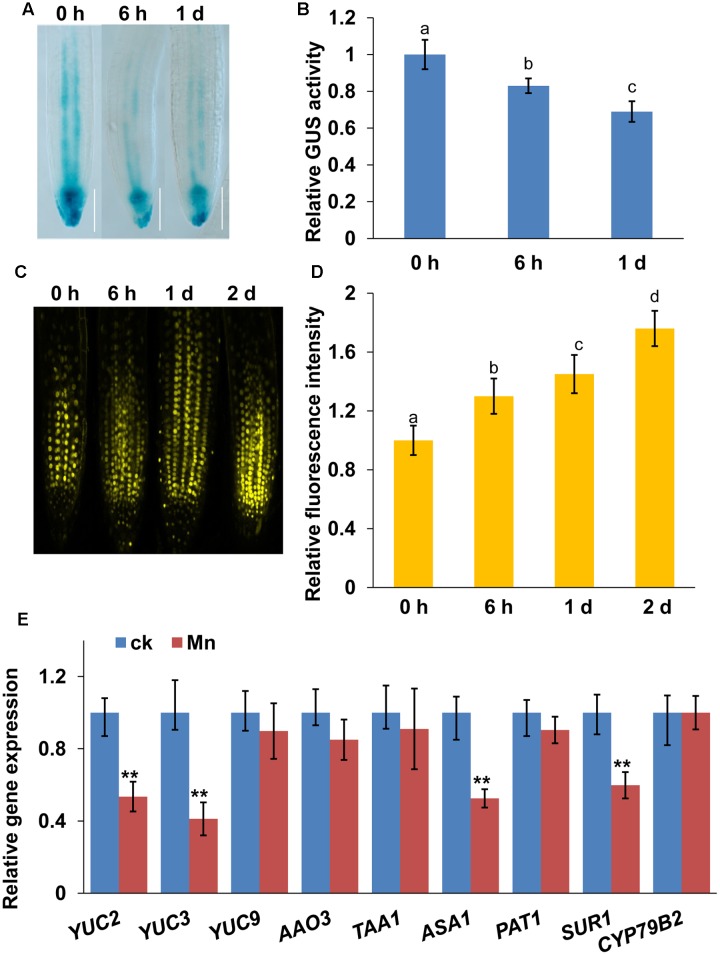
**Manganese toxicity reduces auxin accumulation in root tips.** Image of GUS staining **(A)** and the relative GUS activity **(B)** of 5-day-old *DR5:GUS* seedlings exposed to 4 mM MnCl_2_ for 6 h and 1 day. YFP fluorescence **(C)** and quantification of the *DII-VENUS* fluorescence intensities **(D)** in the roots of 5-day-old *DII-VENUS* seedlings exposed to 4 mM MnCl_2_ for 6 h-2 days. **(E)** Real-time quantitative reverse-transcription polymerase chain reaction (qRT-PCR) analysis of the expression of IAA biosynthesis-related genes in wild-type *col-0* seedlings treated with or without 4 mM MnCl_2_ for 2 days. The expression levels of the indicated genes in the untreated roots were set to 1. The error bars represent the ±SD. Different letters indicate significantly different values (*P* < 0.05 by Tukey’s test). ^∗∗^*P*-value < 0.01.

Auxin plays a central role in regulating root growth and development. Therefore, we examined auxin accumulation in root tips exposed to Mn toxicity using auxin-responsive *DR5:GUS* marker lines, and a auxin-perceptive *DII-VENUS* marker lines, a transgenic line that responds to auxin in a dose-dependent manner without disrupting the activity of the auxin response machinery (Brunoud et al., 2012). Mn toxicity reduced the DR5:GUS fluorescent signal (**Figures 2A,B**), whereas it significantly increased the DII-VENUS fluorescent signal in the root tips (**Figures 2C,D**), suggested that Mn toxicity reduced auxin accumulation in roots.

Next, we examined whether Mn toxicity would also affect the expression of auxin biosynthesis-related genes. First, we examined the transcript levels of auxin biosynthesis-related genes using a quantitative reverse-transcription polymerase chain reaction (qRT-PCR) analysis (**Figure 2E**). Mn toxicity significantly repressed the expression of *YUC2*, *YUC3*, *SUR1*, and *ASA1*, whereas the gene expression of *YUC9*, *AAO3*, *TAA1*, *PAT1*, and *CYP79B2* were unaffected. These data suggested that Mn toxicity repressed the gene expression of auxin biosynthesis-related genes.

### PIN4 and PIN7 are Involved the Mn Toxicity-Induced Inhibition of PR Growth

**FIGURE 3:**
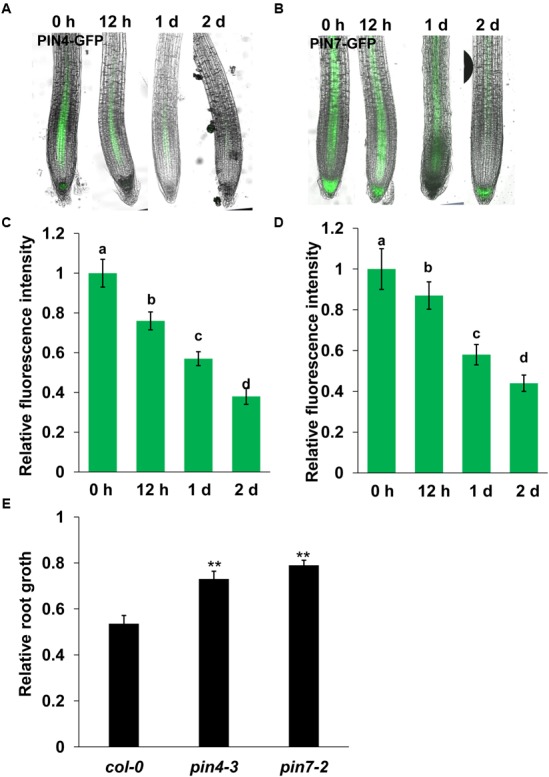
**Manganese toxicity represses the expression of PIN4 and PIN7 in *Arabidopsis* roots.** GFP fluorescence in the roots of 5-day-old *PIN4:GFP* **(A)** or *PIN7:GFP* **(C)** exposed to 4 mM MnCl_2_ for 12–48 h and the quantification of *PIN4:GFP* **(B)** or *PIN7:GFP* **(D)** fluorescence intensities. **(E)** The relative root length of *col-0*, *pin4-3*, and *pin7-2* seedlings treated with 4 mM MnCl_2_ for 2 days compared with untreated seedlings. The error bars represent the ±SD. Different letters indicate significantly different values (*P* < 0.05 by Tukey’s test). ^∗∗^*P*-value < 0.01.

**FIGURE 4:**
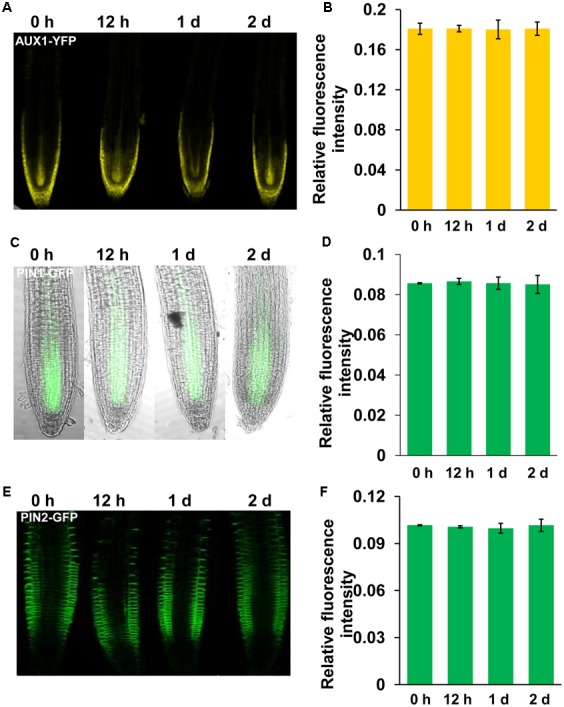
**Manganese toxicity did not affect the expression of AUX1, PIN1, and PIN2 in *Arabidopsis* roots.** YFP/GFP fluorescence in the roots of 5-day-old *AUX1:YFP* **(A)**, *PIN1:GFP* **(C)**, and *PIN2:GFP* **(E)** seedlings exposed to 4 mM MnCl_2_ for 12 h-2 days and quantification of the *AUX1:YFP* **(B)**, *PIN1:GFP* **(D)**, and *PIN2:GFP* **(F)** fluorescence intensities. The error bars represent the ±SD.

Auxin transport plays a role in modulating auxin accumulation and distribution in root tips. We thus wondered whether Mn toxicity-repressed auxin accumulation in root tips is also due to disruption of auxin carrier expression. Thus, we examined the expression of the auxin carriers by using the transgenic lines that express *AUX1:YFP*, *PIN1:GFP*, *PIN2:GFP*, *PIN4:GFP*, and *PIN7:GFP*. We found that Mn treatment significantly reduced the expression of PIN4 (**Figures 3A,B**) and PIN7 (**Figures 3C,D**) in the root tips; however, the expression patterns of AUX1, PIN1, and PIN2 were almost unaffected (**Figure 4**). These data indicated that PIN4 and PIN7 might be involved in Mn-induced inhibition of root growth.

We then investigated the roles of *PIN4* and *PIN7* in the Mn toxicity-induced inhibition of PR growth using *pin4* and *pin7* mutants. We found that both *pin4* and *pin7* mutants showed less inhibition of root growth than *col-0* seedlings (**Figure 3E**). These results indicated that Mn-inhibited PR growth by disrupting the expression of PIN4 and PIN7.

## Discussion

Manganese is an essential micronutrient, but is highly toxic to plant growth and development in excess (Yao et al., 2012). However, the physiological and molecular mechanisms of root growth and development in the presence of excess Mn remain largely unclear. It has been reported that heavy metal stresses, i.e., Cd, Cu, and Fe, disturbed auxin biosynthesis and distribution in root tips, and thereby affected PR growth and LR formation (Yuan et al., 2013; Li et al., 2015; Yuan and Huang, 2016). Auxin is also involved in Al-induced root growth inhibition. The auxin over-producing mutant *yucca* showed increased Al sensitivity (Zhu et al., 2013). In this study, we found that Mn toxicity reduced DR5:GUS expression whereas it increased DII-VENUS fluorescent signal in the root tips, indicating that exposure to excess Mn resulted in PR growth inhibition by reducing auxin accumulation in root tips.

Our results indicated that Mn toxicity decreased auxin accumulation in roots by reducing auxin biosynthesis and repressing auxin transport via the decrease of the expression of auxin efflux carriers PIN4 and PIN7. Several lines of evidence support these conclusions. (1) qRT-PCR analysis indicated that four auxin biosynthesis-related genes (*YUC2*, *YUC3*, *ASA1*, and *SUR1*) were markedly downregulated in response to Mn toxicity. (2) Mn toxicity repressed the protein expression of PIN4 and PIN7, as indicated by PIN4-GFP and PIN7-GFP fluorescence. (3) Genetic analysis supported the result in *pin4* and *pin7* mutants.

Proper auxin accumulation depends on the coordination between auxin transport and biosynthesis (Li et al., 2015; Liu et al., 2015, 2016). PIN proteins are the central rate-limiting components that regulate auxin transport and optimal auxin accumulation in the root tips (Petrasek and Friml, 2009; Yuan and Huang, 2016). The effects of heavy metal toxicity on auxin carrier expression and the promotion of root growth repression has been widely reported (Yuan et al., 2013; Li et al., 2015; Yuan and Huang, 2016). PIN1 is involved in Cu toxicity-induced PR growth inhibition (Yuan et al., 2013). Yuan and Huang (2016) found that PIN1/3/7 are involved in Cd-repressed auxin accumulation in root tips. Li et al. (2015) found that AUX1 and PIN2 are involved in Fe toxicity-mediated RSA remodeling. Yang et al. (2014) found that Al toxicity specifically induced auxin accumulation in the TZ of the root tips. Al toxicity affected the expression and localization of PIN2 in roots, and thereby inhibiting root growth (Shen et al., 2008; Wu et al., 2014). Although different auxin efflux carriers functioned in distinct heavy metal stresses, the changes in PIN protein levels were a common mechanisms underlying the heavy metal-induced root growth inhibition (Yuan and Huang, 2016). In this study, we found that Mn toxicity decreased both PIN4-GFP and PIN7-GFP fluorescence, suggesting clear roles for PIN4 and PIN7 in Mn-repressed auxin accumulation in root tips. Both PIN4 and PIN7 are required for PAT from shoots to roots and maintaining a maximal auxin concentration in the quiescent center (QC) and a steep auxin gradient in the proximal meristem (Friml et al., 2003; Laskowski et al., 2008; Liu et al., 2016). The *pin7* mutant failed to establish the apical-basal auxin gradient in embryos (Friml et al., 2003). The *pin4* mutant accumulates higher auxin levels in root tips (Friml et al., 2002). Both the *pin4* and *pin7* mutants exhibited less sensitivity to Mn toxicity, which suggested that PIN4 and PIN7 mediate Mn toxicity-reduced auxin accumulation and subsequently inhibited-PR elongation.

Root meristematic cell division potential is an important factor that affect root growth (Liu et al., 2016; Yuan and Huang, 2016). Root meristem activity is controlled by auxin and cytokinin, and their interaction (Ioio et al., 2008). Auxin controls meristem growth and cell division by mediating degradation of SHY2 protein, a repressor of auxin signaling. Mn toxicity decreased auxin accumulation in roots, and thereby reducing meristematic cell division in root tips, as indicated by the *pCYCB1;1: CYCB1;1-GUS* marker line. Although Mn toxicity reduced the GUS activity in the roots of *CYCB1;1:GUS* seedlings, the GUS expression is still kept in the most intensively dividing cells of the meristematic zone, suggesting that Mn toxicity reduced root meristematic cell activity, but it did not completely induce cell death in the zone.

In summary, our study indicate that Mn toxicity inhibited PR growth by reducing auxin biosynthesis and repressing the expression of auxin efflux transporters PIN4 and PIN7 to reduce auxin levels in root tips, resulting in reduced root meristematic cell division.

## Author Contributions

JX and XP conceived the study and designed the experiments. JZ, PZ, and RW carried out the experiments. JX, JZ, WW, HZ, and HW analyzed the data. JX, JZ, WW, XP, and HZ wrote the manuscript.

## Conflict of Interest Statement

The authors declare that the research was conducted in the absence of any commercial or financial relationships that could be construed as a potential conflict of interest.
