# Supplementary material for: Manganese Toxicity Inhibited Root Growth by Disrupting Auxin Biosynthesis and Transport in Arabidopsis
Source: Front Plant Sci. 2017 Mar 3;8:272. doi: 10.3389/fpls.2017.00272 (PMC5334637; doi:10.3389/fpls.2017.00272)
Supplement: Supplementary file 1 [file Table_1.DOCX]

**Supplementary file**

**Table S1.** List of the primers for qRT-PCR analysis of the genes.

| **Gene** | **Primers** |
| --- | --- |
| ***AtYUC2*** | 5’GGTGACACGGATCGGTTAGGGT3’  5’TGCCGAATAATGCATTACCCGT3’ |
| ***AtYUC3*** | 5’CTTGAGATTGATTCCGTTATTC3  5’GGAGAAGAAGTCGTTGTC3’ |
| ***AtYUC9*** | 5’ATCTTGCTAACCACAATG3’  5’CCACTTCATCATCATCAC3 |
| ***AtSUR1*** | 5’GACCACCAAGGTGTTACAATCC3’  5’ATTATTGTGGCAGGGTCAGG3’ |
| ***AtAAO3*** | 5’GGAGTCAGCGAGGTGGAAGT3’  5’TGCTCCTTCGGTCTGTCCTAA3’ |
| ***AtASA1*** | 5’ATGTCTTCCTCTATGAACGTAGC3’  5’ACAGCGGTAAATTGGTATAAGG3’ |
| ***AtPAT1*** | 5’ATGGTTATTGCGGTGGCGAC3’  5’ATCGTCGCCGACTCAATGTC3’ |
| ***AtTAA1*** | 5’CTCCAAGATCACAGGCCACGCTGGG3’  5’GACTCCTTAGACACACCAATCGAGTTC3’ |
| ***AtCYP79B2*** | 5’CACGATGATGCTCGCGAGACT3’  5’TCACTTCACCGTCGGGTAGAGA3’ |
